# Terahertz metamaterials for light-driven magnetism

**DOI:** 10.1515/nanoph-2023-0801

**Published:** 2024-02-02

**Authors:** Matteo Pancaldi, Paolo Vavassori, Stefano Bonetti

**Affiliations:** Department of Molecular Sciences and Nanosystems, Ca’ Foscari University of Venice, 30172 Venezia Mestre, Italy; CIC nanoGUNE BRTA, 20018 Donostia-San Sebastián, Spain; IKERBASQUE, Basque Foundation for Science, 48013 Bilbao, Spain; Department of Physics, Stockholm University, 10691 Stockholm, Sweden

**Keywords:** terahertz fields, terahertz metamaterials, ultrafast magnetism

## Abstract

We describe the design of two types of metamaterials aimed at enhancing terahertz field pulses that can be used to control the magnetic state in condensed matter systems. The first structure is a so-called “dragonfly” antenna, able to realize a five-fold enhancement of the impinging terahertz magnetic field, while preserving its broadband features. For currently available state-of-the-art table top sources, this leads to peak magnetic fields exceeding 1 T. The second structure is an octopole antenna aimed at enhancing a circularly-polarized terahertz electric field, while preserving its polarization state. We obtain a five-fold enhancement of the electric field, hence expected to exceed the 1 MV/cm peak amplitude. Both our structures can be readily fabricated on top of virtually any material.

## 1 Introduction

The condensed matter physics community has recently shown an increased interest in the use of electromagnetic radiation in the terahertz range for the study of material systems [1]. For example, the field of ultrafast magnetism was born almost three decades ago [2], but it is only in the last decade that intense terahertz radiation has been introduced in the game [3], [4], [5], [6], [7]. A major difference between more conventional near-infrared and terahertz pulses is that, in the latter case, the magnetic field component is slow enough to allow for a relatively strong Zeeman torque on the magnetization. In turn, this enables coherent control of the magnetization dynamics, in contrast to visible/near-infrared radiation where incoherent thermalization of non-equilibrium electron states is the dominant effect.

Intense terahertz pulses can be obtained both at large-scale facilities [8], [9], [10], [11], [12], as well as in table-top setups [13], [14], [15], [16]. The latter are enabling the widespread use of single-cycle (or few-cycles) terahertz radiation to probe and control matter [1], [17], [18], [19]. However, in order to fully explore dynamical regimes beyond the linear one, there is a need for terahertz pulses of even larger amplitude than what achievable at the source. To this end, much effort has been dedicated to the design of metamaterial structures, i.e. patterned metallic thin-film antennas, aimed at locally enhancing the linearly-polarized terahertz electric field [20], [21], [22], [23]. Surprisingly, only a few studies have been performed with the goal of enhancing the terahertz magnetic field component [24], [25], [26], [27], which is of relevance (e.g.) for the study of nonlinear magnetization dynamics [28], and for exploring new magnetization switching regimes driven by spin inertia [29], [30]. However, magnetic fields are not the only way for interacting with the magnetic properties of matter. High-amplitude electric fields at terahertz frequencies allow for the direct excitation of collective lattice modes [17]. In the case of perpendicularly degenerate or chiral phonons, it is possible to drive atoms into circular motion, which has been shown to be associated with an effective magnetic field acting on the spins and affecting the magnetization [31], [32], [33], [34]. The efficient control of this phenomenon on ultrafast time scales requires high-field circularly-polarized terahertz pump pulses, but little attention has been paid towards designing metamaterials for enhancing terahertz fields with non-linear polarization.

In this article, we present the design, using three-dimensional finite element simulations, of two metamaterial structures aimed at filling these voids. For the first design, after revising the current approaches for enhancing the magnetic field component (mainly split-ring resonators), we present a “dragonfly” antenna which combines two different structures: a bow-tie broadband antenna [35] and a coplanar stripline [36]. The bow-tie antenna is the broadband receiving part, whereas the stripline transmits the signal in the region of interest, and it is responsible for the enhancement of the terahertz magnetic field. Such a design mostly preserves the broadband nature of the incoming terahertz field. For the second design, we build upon a strategy developed for radiation in the visible/near-infrared range [37], [38], and apply it to the terahertz range to obtain a metamaterial able to enhance circularly-polarized electric fields [39]. Both proposed structures have dimensions in the tens of micrometer range, and can be readily fabricated using standard lithographic and deposition techniques.

## 2 The dragonfly antenna

Current structures devoted to the enhancement of the magnetic field are based on the split-ring resonator geometry, where electromagnetic radiation at normal incidence can induce a current when the electric field is oriented perpendicularly to the gap [40], [41], [42]. In turn, the current generates a magnetic field normal to the plane of the split-ring, whose amplitude is enhanced with respect to the magnetic field component of the incoming radiation. Since the enhancement is local, i.e. confined inside the split-ring, such structures have to be patterned in close proximity to the magnetic feature of interest [24], [25], [43], [44]. As a drawback, the split-ring design lacks flexibility, and its frequency response is characterized by narrow peaks in correspondence of the resonance modes. Through an equivalent circuit model, it is possible to recognize that the split-ring behaves as a LC circuit, and the magnetic resonance frequency is inversely proportional to the split-ring area [45], [46]. The resonance tuning is then performed by varying the ring diameter, which also influences the extent of the field enhancement.

In order to decouple the resonance frequency from the field enhancement, a modified geometry can be considered, as shown by Polley et al. [26] with the so-called “question mark” (QM) metamaterial. When a straight section is added to the split-ring, and the incident field is linearly polarized along that section, the overall electrical length can be changed without altering the diameter of the ring. By doing so, the active region can maintain a constant size, independently from the target resonance frequency. However, such a structure still possesses narrow resonances, which can in principle be an unwanted feature when working with broadband pulses, and the impulsive characteristics of the excitation need to be preserved.

**Figure 1:**
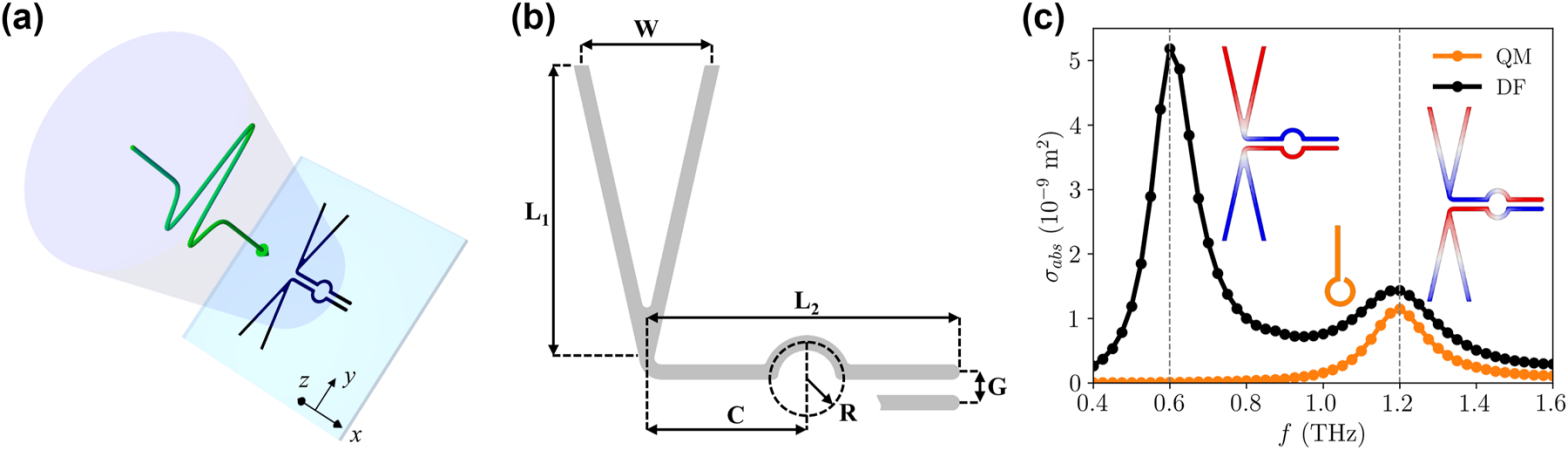
Scheme of the DF antenna and absorption cross section. (a) Scheme of the DF antenna stimulated by a linearly-polarized (along the *y*-axis) quasi-single-cycle terahertz pulse. The *z* = 0 plane represents the air/substrate interface. (b) Design of the gold DF antenna, for pointing out the relevant parameters: *W* = 30 μm, *L*_1_ = *L*_2_ = 60 μm, *C* = 30 μm, *R* = 7.5 μm, and *G* = 6 μm, with a track width of 3 μm and a thickness of 100 nm. (c) Absorption cross sections calculated in the frequency domain for linearly polarized radiation at normal incidence, for both QM and DF designs. The insets show the normalized charge distribution (in the $\left[-1,1\right]$ range) of the oscillation modes corresponding to the absorption peaks for the DF antenna, and a scheme of the QM antenna, whose parameters were tuned to show a resonance at 1.2 THz. For that peak, the full width at half maximum for the (benchmark) QM antenna is 0.16 THz, while it is 0.21 THz for the DF antenna.

Here, we propose a design for increasing the bandwidth and preserve the frequency content of short terahertz pulses, by combining complementary geometries in the “dragonfly” (DF) design, as schematically shown in Figure 1(a) and (b). A bow-tie antenna [35], [47], [48] constitutes the broadband receiving part (characterized by the *W* and *L*_1_ dimensions), which is efficiently stimulated when aligned along the polarization axis of the impinging terahertz radiation (*y*-axis in the scheme). The electromagnetic field is then transmitted to the circular active region of radius *R* via a coplanar stripline [36], [49], [50] of length *L*_2_ and pitch *G*. Our design aligns with the quest for metamaterials characterized by broadband electromagnetic functionalities, as reviewed in Ref. [51].

The electromagnetic properties of the DF antenna have been verified by means of finite element simulations in the frequency domain [52], with the relevant dimensions specified in the caption of Figure 1(b). A detailed drawing of the considered DF geometry is shown in the Supplementary Material, Figure S1(a). A gold antenna, with a thickness of 100 nm, was placed on top of a semi-infinite crystalline quartz substrate. The material properties of gold were specified in terms of the electrical conductivity *σ* = 4.09⋅10^7^ S/m [53], while the substrate was characterized by a refractive index *n*_subs_ = 2.1 (considered constant in the analyzed frequency range) [54]. The other semi-infinite domain, where the incident wave comes from, is considered to be air, with a refractive index *n*_air_ = 1. Further details on the simulation strategy are reported in the Supplementary Material, Section S2. The antenna’s dimensions have been chosen to work in a frequency range compatible with OH1, an organic crystal used for obtaining high-amplitude terahertz fields in table-top setups via optical rectification [55].

As a first characterization, Figure 1(c) shows the absorption cross section, which is the ratio between the power absorbed by the antenna and the intensity of the incoming electromagnetic field. For the DF design, the absorption cross section shows two peaks in the considered frequency range. The charge distributions shown in the insets allow identifying the nature of the resonance modes: the first peak corresponds to the half-dipole resonance at 0.6 THz, while the second peak corresponds to the dipole resonance at 1.2 THz [56]. In both cases, the current flows in opposite directions in the two halves of the coplanar stripline, so determining an effective current loop in the circular active region. Besides being associated with a lower absorption, and hence with a lower average current, the active region lies close to the zeros of the surface charge for the 1.2 THz resonance, so a higher peak current can be obtained with respect to the 0.6 THz resonance. As a reference, Figure 1(c) also shows the absorption cross section for a gold QM antenna (100 nm thick), whose dimensions were selected in order to have the same resonance frequency and a similar active region area, as shown in the Supplementary Material, Figure S1(b). A comparison of the peak full width at half maximum for both geometries (0.16 THz for the QM, 0.21 THz for the DF) proves that the DF antenna possesses a larger bandwidth around the working frequency. For both structures, the full width at half maximum was extracted by performing a Gaussian fit of the peak. However, in the case of the DF antenna, we also added a quadratic background for fitting the absorption cross section in the $\left[0.9,1.6\right]$ THz range, in order to have a better comparison with the QM peak.

**Figure 2:**
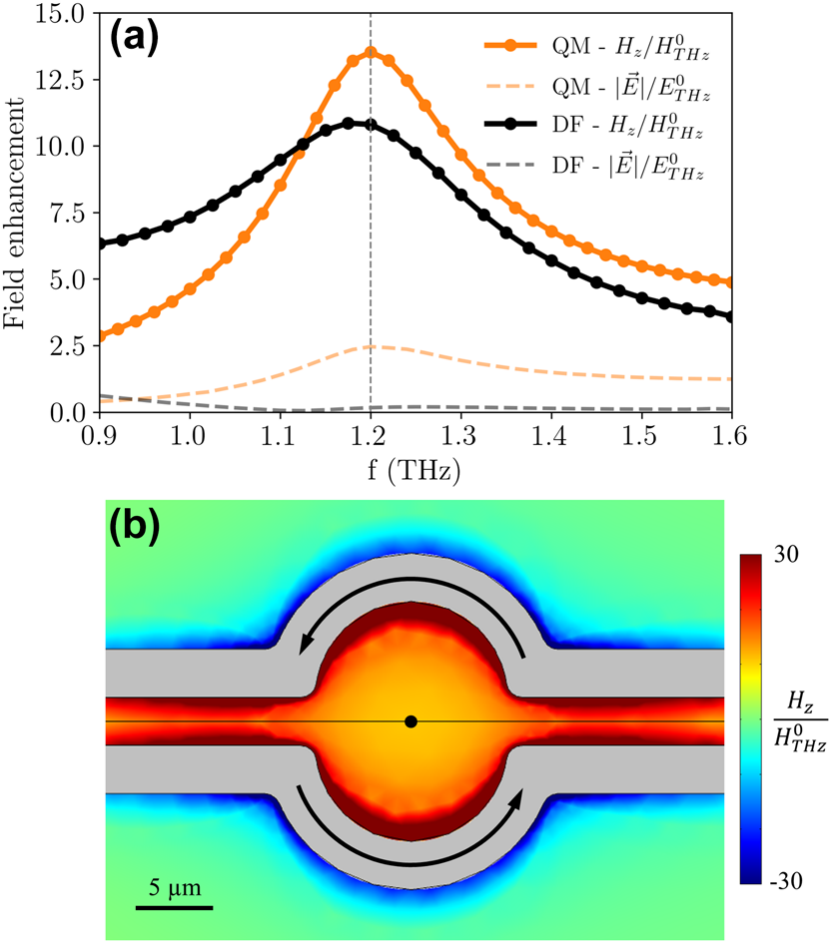
Field enhancement in the frequency domain. (a) Field enhancement at the center of the active region calculated in the frequency domain with respect to the incident field ( $E_{\text{THz}}^{0}$ , $H_{\text{THz}}^{0}$ ). Both the *z*-component of the magnetic field (*H*_*z*_) and the magnitude of the electric field $\left(\left|\vec{E}\right|\right)$ are shown. (b) Map of the out-of-plane magnetic field enhancement for the DF antenna at 1.2 THz in the *z* = 0 plane. The arrows indicate the direction of the current in the coplanar stripline for generating a *H*_*z*_ component in the positive *z*-axis direction.

A comparison of the *H*_*z*_ field enhancement at the center of the active region is reported in Figure 2(a). The enhancement is calculated with respect to the incident electromagnetic field amplitude. At 1.2 THz, both geometries show a comparable magnetic field enhancement (13.5 for the QM, 10.8 for the DF), but the DF antenna is associated to a flatter frequency response in the considered range, which is a consequence of the enhanced peak bandwidth and to the presence of the other resonance mode at 0.6 THz [57]. Moreover, due to its symmetry, the DF antenna is capable of enhancing the out-of-plane magnetic field while suppressing the electric field. This feature is relevant when it is important to decouple the two fields, which is not possible for a propagating electromagnetic wave, but it is feasible in the quasi-static regime [58]. As shown by the black dashed line in Figure 2(a), the electric field in the DF geometry is weakened with respect to the electric field component of the incident field. A map of the electric field enhancement in the *z* = 0 plane is reported in the Supplementary Material, Figure S2(c). As a figure of merit, we introduce the ratio between the magnetic and electric field amplitudes, normalized to the impedance of free space (*Z*_0_). For an electromagnetic wave traveling in vacuum (as the incident radiation in the considered simulations), we have that *Z*_0_ *H*/*E* = 1. At 1.2 THz, in the center of the active region we obtain that *Z*_0_ *H*/*E* = 65.3 for the DF antenna, while *Z*_0_ *H*/*E* = 5.5 for the QM antenna, showing that this latter geometry is less effective in shielding the active region from the incident electric field. The symmetry of the DF antenna is also relevant when considering the magnetic field enhancement map shown in Figure 2(b). The enhancement grows uniformly from the center to the edge of the circular active region, and it is also present in the gap between the linear sections of the coplanar stripline (similarly to a pair of wires carrying opposite currents). To show the decay of the enhancement along the out-of-plane direction, a map in a plane parallel to the *yz*-plane and passing through the center of the active region is reported in the Supplementary Material, Figure S2(a). From the map, a line profile parallel to the *z*-axis can be extracted (Figure S2(b)), and it is possible to see that the decay of the out-of-plane magnetic field component closely follows the decay of the magnetic field generated by a circular current loop, further validating the design of the antenna.

**Figure 3:**
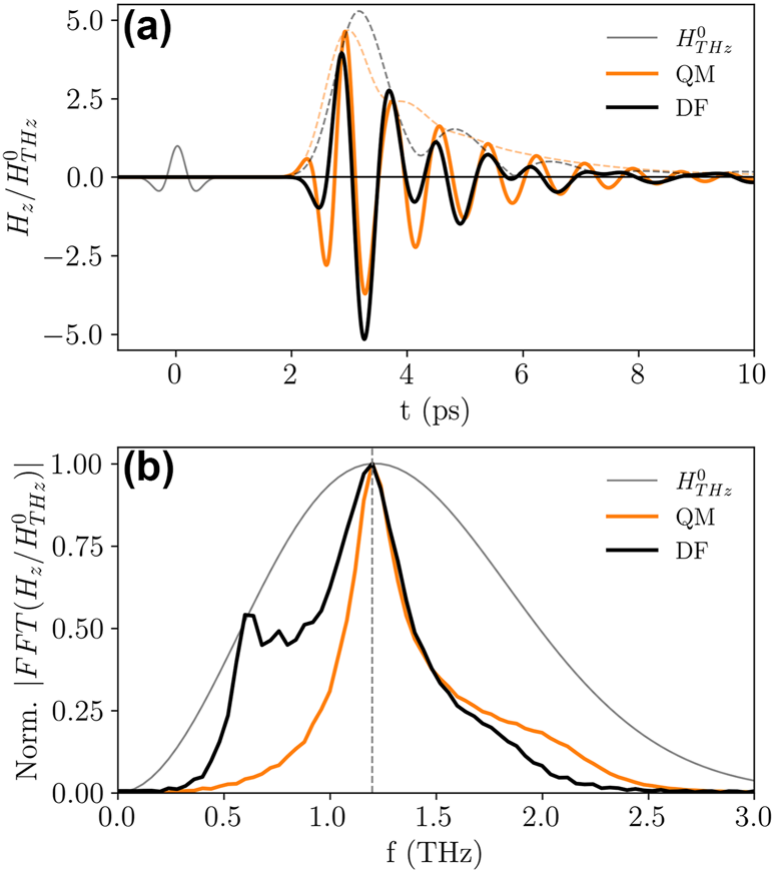
Field enhancement in the time domain. (a) Magnetic field enhancement at the center of the active region calculated in the time domain considering a quasi-single-cycle terahertz pump pulse (modeled as the second derivative of a Gaussian-shaped pulse). The dashed lines represent the signals’ envelopes, indicating a faster decay for the DF geometry. (b) Normalized FFT amplitudes for the signals in panel (a). The wider bandwidth of the DF antenna helps in better preserving the temporal profile of the incident pulse.

Finally, the effect of a broadband terahertz pulse has been simulated in the time domain, in order to corroborate all the results obtained in the frequency domain and to take into account a terahertz pulse with a realistic frequency spectrum [55]. As shown in Figure 3(a), the incident quasi-single-cycle terahertz pulse was described considering the second derivative of a Gaussian-shaped pulse [13] with a standard deviation of 0.185 ps, as indicated in the Supplementary Material, Eq. (S2). Figure 3(a) also shows the *H*_*z*_ enhancement as a function of time at the center of the active region: for both the DF and QM antennas, a five-fold enhancement can be obtained. Since the incident pulse has a broad spectrum, as reported in Figure 3(b), the maximum enhancement over the whole incident pulse bandwidth is reduced with respect to the resonant enhancement shown in Figure 2(a). However, for a state-of-the-art quasi-single-cycle terahertz magnetic field pulse with an amplitude of 300 mT, peak magnetic fields of the order of 1.5 T can be reached, which can be utilized (e.g.) for triggering non-linear magnetization dynamics in the few-ps time scale [28]. Moreover, by looking at the signals’ envelopes highlighted by the dashed lines in Figure 3(a), it is possible to notice that the oscillations following the main pulse (i.e. after 4 ps) have a faster decay for the DF antenna with respect to the QM. Those oscillations are a consequence of the filtering effect caused by the antenna bandwidth, which is less pronounced for the DF geometry, so confirming the increased bandwidth of this design. A comparison of the bandwidth for both designs can be obtained in the time domain by extracting the amplitude of the Fourier transform (via FFT), as shown in Figure 3(b). Again, the wider resonance peak at 1.2 THz and the presence of the 0.6 THz peak make the DF design better suited for preserving the impulsive characteristics of the pump pulse.

## 3 The octopole antenna

Another situation of recent growing interest for the study of light-driven magnetism is the one of enhancing the terahertz electric field while preserving the circular polarization of the excitation. Historically, circularly-polarized light has often been used for investigating magnetism in materials. Most prominent is the case of the magnetic circular dichroism at X-ray or extreme UV wavelengths, which exploits the different absorption coefficient of light with opposite helicity to quantitatively retrieve the spin and orbital contribution to the overall magnetic moment of a sample [59]. This effect is present and has been used even in the optical regime, although quantitative material properties are not easily extracted in that case. Going further down in the photon energy, reaching the terahertz range, circularly-polarized light has been recently employed not to probe, but rather to drive novel magnetic states in matter. As representative example, we cite the dynamical multiferroicity effect in diamagnetic insulators [60], which has been recently shown to be enhanced by the Barnett effect in two different experiments, leading to large magnetic moments driven by circularly-polarized terahertz fields [61], [62]. It seems therefore timely to design metamaterials capable of enhancing such fields, which are anticipated to allow for enhanced control of magnetic states in matter.

**Figure 4:**
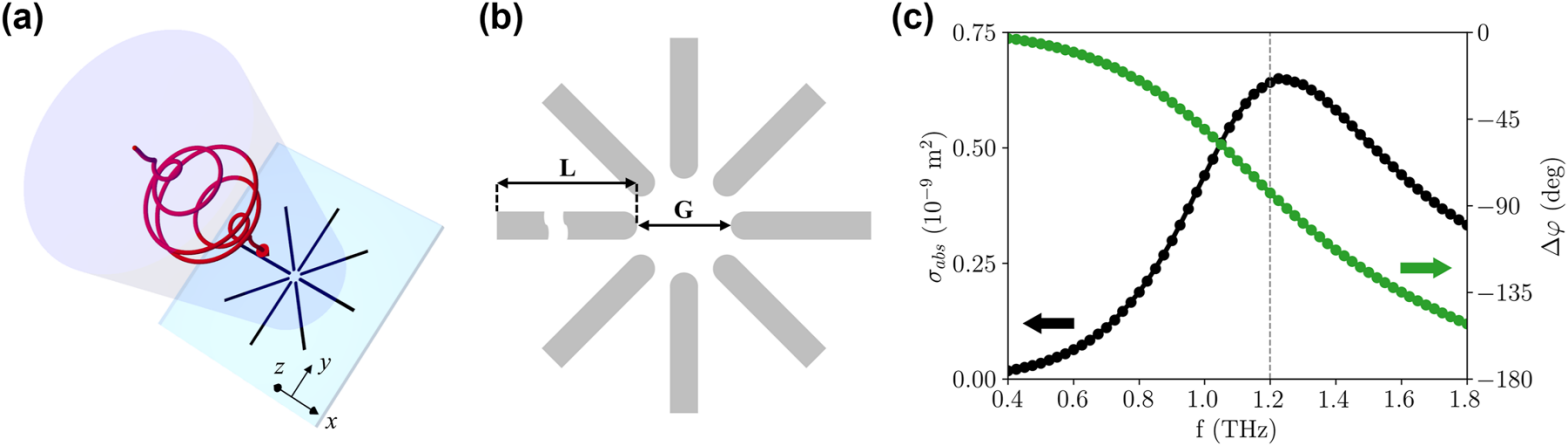
Scheme of the octopole antenna and absorption cross section. (a) Scheme of the octopole antenna stimulated by a circularly-polarized narrowband terahertz pulse. The *z* = 0 plane represents the air/substrate interface. (b) Design of the gold octopole antenna, for pointing out the relevant parameters: *L* = 65 μm, and *G* = 10 μm, with a bar width of 3 μm and a thickness of 100 nm. (c) Absorption cross section calculated in the frequency domain for circularly-polarized radiation at normal incidence. The plot also shows the relative phase, which is evaluated between the incident field and the field scattered by the antenna.

The design that we present for this case is a variation of the cross antenna proposed in Refs. [37], [39], where we add two additional pairs of dipole antennas along the diagonals, as depicted in Figure 4(a). We name this geometry the “octopole” antenna, for obvious reasons. Each of the electrodes of the antenna, of length *L*, is placed at an angle of 45° with respect to the nearest neighboring one, creating a gap *G* between opposite electrodes, as shown in Figure 4(b). The electrodes’ edges close to the central active regions are rounded, in order to increase the field uniformity. Through finite element simulations in the frequency domain [52], a 100 nm-thick gold octopole antenna was designed to resonate at a frequency of 1.2 THz, as indicated by the absorption cross section in Figure 4(c) calculated for circularly-polarized radiation at normal incidence. The figure shows an absorption peak at the design frequency and, consistently, a 90° phase difference Δ*φ* of the scattered radiation as compared to the incident field. All the material parameters considered in the simulations are the same as the ones presented in Section 2 for the DF antenna, and further details on the simulation strategy are reported in the Supplementary Material, Section S2.

**Figure 5:**
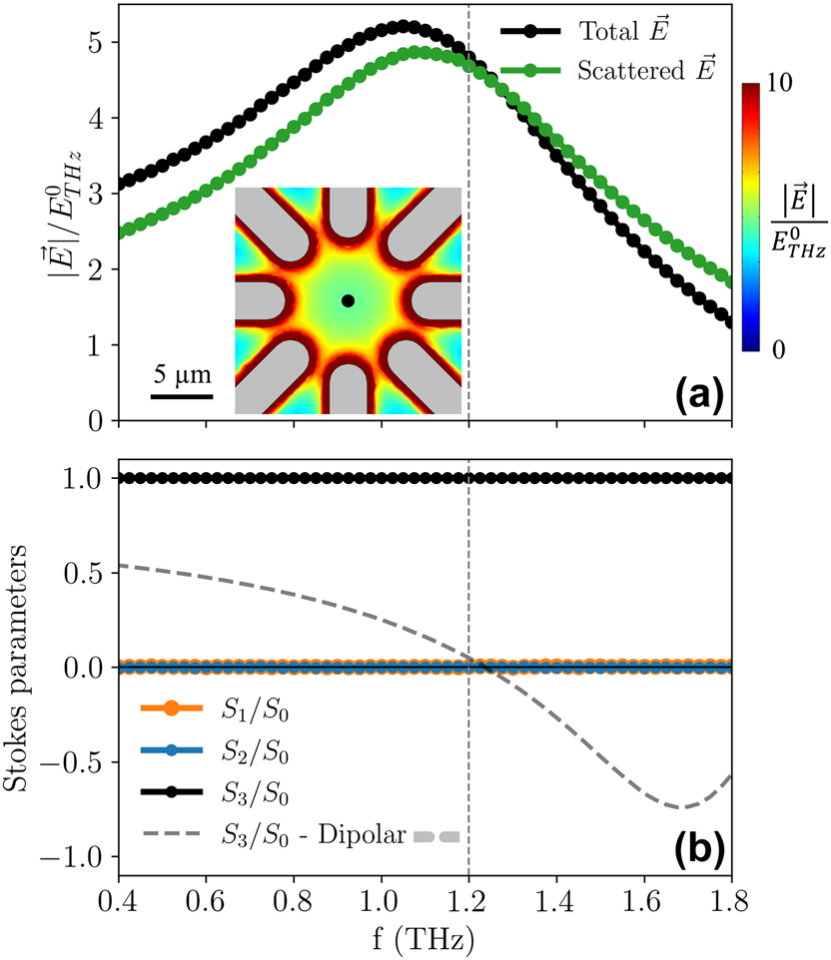
Field enhancement and Stokes parameters in the frequency domain. (a) Electric field enhancement at the center of the active region calculated in the frequency domain with respect to the incident field $E_{\text{THz}}^{0}$ . The black curve represents the total electric field, while the green curve only considers the electric field scattered by the metamaterial. The inset shows a map of the electric field enhancement for the *z* = 0 plane at 1.2 THz in the central part of the metamaterial. (b) Normalized Stokes parameters as a function of frequency, for quantifying the polarization state in the center of the active region. As a comparison, the dashed line shows the *S*_3_/*S*_0_ parameter for a dipole antenna.

Figure 5(a) shows the electric field enhancement (both the total one, in black, and the scattered one, in green) in the center of the octopole antenna. The difference between the total and the scattered field is determined by the resonator phase: at frequencies below resonance, the electric field generated by the antenna mainly adds in phase to the incident electric field, while above resonance the opposite is true, since the two contributions are progressively becoming out of phase. In any case, the simulated data indicate an approximate five-fold enhancement in the frequency range between 1 and 1.2 THz. For a state-of-the-art narrowband terahertz electric field pulse of 200 kV/cm amplitude, this would mean that MV/cm electric fields are achievable. The two-dimensional map in the inset of Figure 5(a) reveals that the enhancement at 1.2 THz is rather uniform in a central circular area of diameter *G*/2, and the uniformity is improved by rounding the end of the electrodes composing the metamaterial. A map of the enhancement in a plane parallel to the *xz*-plane and passing through the center of the active region is reported in the Supplementary Material, Figure S3(a). The performance of the octopole antenna can also be compared to the standard cross antenna [37], [39], where only two orthogonal pairs of dipole antennas are active. As shown in the Supplementary Material, Figure S4, the main benefits of the octopole design consist in the enhancement of the antenna bandwidth, and in the higher field uniformity in the central circular area of diameter *G*/2.

In order to characterize the degree of polarization in the octopole antenna after exciting it with circularly-polarized terahertz radiation, we calculated the Stokes parameters *S*_1_, *S*_2_ and *S*_3_, and normalize them to *S*_0_. From the Stokes parameters, a full analysis of the polarization state can be obtained [63]: while the *S*_0_ parameter represents the total intensity, *S*_1_ and *S*_2_ are associated with linear polarization along two sets of orthogonal axes, and *S*_3_ is associated with circular polarization. The results are shown in Figure 5(b). The simulations show a perfect degree of circular polarization at the center of the metamaterial, with the *S*_3_/*S*_0_ ratio being equal to 1 in the range from 0.4 to 1.8 THz, where *S*_1_/*S*_0_ and *S*_2_/*S*_0_ are negligible. Such a behavior can be compared with the *S*_3_/*S*_0_ ratio calculated for a dipole antenna [20], [22] with the same *L* and *G* dimensions used for the octopole. In such a case, the circular polarization is totally lost at the resonance frequency, and it shows a non-monotonous trend over the same range. Maps of the normalized Stokes parameters at 1.2 THz as a function of space in the active region are available in the Supplementary Material, Figure S3(f)–(h), which show that the polarization in most of the active region area is predominantly circular.

## 4 Conclusions

We designed two novel terahertz metamaterials aimed at filling up the void left by conventional structures of this kind. In the first design, we proposed a “dragonfly” structure able to enhance the terahertz magnetic field of a broadband, single-cycle pulse in the out-of-plane direction. Our design greatly extends the overall bandwidth of enhancement, resulting in a five-fold peak enhancement in the time domain, uniform over an area of several micrometers in lateral size. This would allow for quasi-single-cycle local magnetic field pulses with amplitude of more than 1 T. We anticipate that such intense and short magnetic fields will open up for the study of nonlinear magnetization dynamics in several magnetic systems, including antiferromagnets and altermagnets [64]. In the second design, we suggested an octopole antenna design able to reach a five-fold enhancement of an incident narrowband, circularly-polarized terahertz electric field, retaining its polarization state uniformly over an area with lateral size of several micrometers in this case as well. Our design allows for reaching the MV/cm electric field amplitude regime, a regime where nonlinear effects become evident. For the case of insulating materials, the implementation of our structures in experiments is particularly straightforward, as they can be directly patterned on top of the target material. On the other hand, for a metallic material, a metallic antenna cannot be directly fabricated on top of it, but an insulating spacer is needed. The optimal spacer thickness and the role of a continuous conductive surface below the antenna can be evaluated by extending the simulations presented in this work. However, in the case of nanostructures (of any material), they can be conveniently patterned/deposited inside the active region, making the presented metamaterials the ideal support devices for nanophotonic experiments.

## Supplementary Material

Supplementary Material Details
