# Supplementary material for: Terahertz metamaterials for light-driven magnetism
Source: Nanophotonics. 2024 Feb 2;13(10):1891–8. doi: 10.1515/nanoph-2023-0801 (PMC11501536; doi:10.1515/nanoph-2023-0801)
Supplement: Supplementary file 1 — Supplementary Material Details [file j_nanoph-2023-0801_suppl_001.pdf]

Supplementary Material for "Terahertz metamaterials for light-driven magnetism"

Matteo Pancaldi[1,*], Paolo Vavassori[2,3], and Stefano Bonetti[1,4,*]

[1] *Dept. of Molecular Sciences and Nanosystems, Ca' Foscari University of Venice, 30172 Mestre, Italy.*
[2] *CIC nanoGUNE BRTA, 20018 Donostia-San Sebastián, Spain.*
[3] *IKERBASQUE, Basque Foundation for Science, 48013 Bilbao, Spain.*
[4] *Department of Physics, Stockholm University, 10691 Stockholm, Sweden.*
[*] *matteo.pancaldi@unive.it; stefano.bonetti@unive.it*

S1 Drawings of the dragonfly and question mark antennas

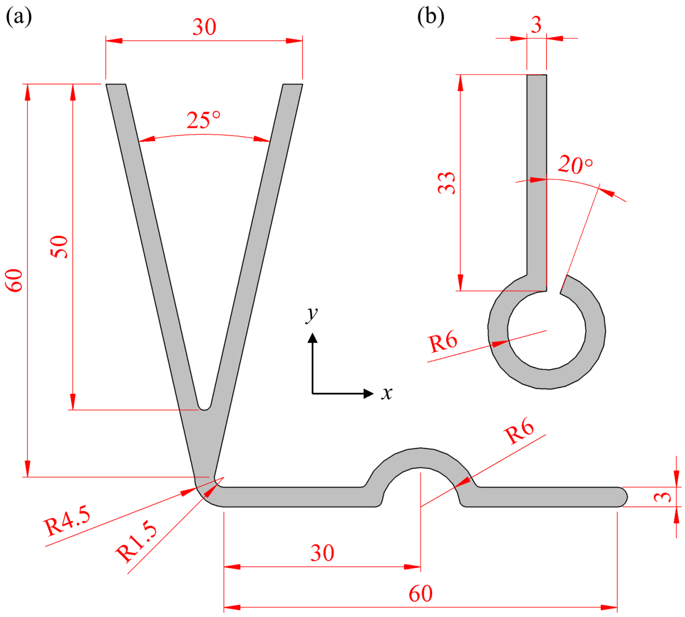

Figure S1: Drawings of (a) the dragonfly and (b) the question mark antennas, with all the linear dimensions shown in micrometers. The letter "R" indicates the radius. For both geometries, the incident terahertz electric field is linearly polarized along the y-axis direction.

S2 Finite element simulations with COMSOL

The electromagnetic properties of the presented antennas have been verified by means of three-dimensional finite element simulations performed with the COMSOL Multiphysics software [1]. For the frequency-domain simulations, we made use of the "Electromagnetic Waves, Frequency Domain" physics interface available in the "Wave Optics" module, which solves for the spatial distribution of a time-harmonic electromagnetic field, assuming an $\exp(i\omega t)$ time dependence. In particular, we worked in the framework of the "scattered field" formulation, where a known background field at the interface between two semi-infinite media (air, with $n_{air} = 1$, and crystalline quartz) becomes a source term in Maxwell's equations, and only the electromagnetic field generated in the presence of the antenna is solved for. The use of semi-infinite media helps in neglecting the effect of multiple reflections in a finite-thickness substrate, which are not relevant for quasi-single-cycle terahertz pulses when the substrate thickness is much greater than half of the peak wavelength in the material. In other words, if the substrate is too thick, multiple reflections cannot interfere. However, for thin substrates the actual thickness has to be taken into account in the antenna design process, as discussed in the case of a 100 μm-thick GaP slab by Savoini et al. [2]. To model the air/crystalline quartz interface, we considered a spherical domain divided in two parts by the $z = 0$ plane, and surrounded by perfectly matched layers for avoiding unwanted reflections at the external model boundaries [3]. The crystalline quartz substrate was characterized by a refractive index $n_{subs} = 2.1$ (constant in the analyzed frequency range) [4], and a 100 nm-thick gold antenna was placed on top of it. The material properties of gold were specified in terms of the electrical conductivity $\sigma = 4.09 \cdot 10^7$ S/m [5], which is related to a frequency-dependent permittivity $\varepsilon(\omega)$ via [6]

$$\varepsilon(\omega) = \varepsilon_0 \left(1 - i\frac{\sigma}{\omega\varepsilon_0}\right), \tag{S1}$$

where ε_0 is the vacuum permittivity. To correctly capture the field variations in proximity of the antenna, the antenna was enclosed in a cylindrical domain with thickness of 20 μm and radius of 100 μm. Such auxiliary domain allowed us reducing the size of the tetrahedral mesh elements where needed, keeping a coarser mesh in the rest of the spherical domain (with a maximum linear size of 1/8 of the wavelength). To maintain an approximately constant number of mesh elements for each considered wavelength (which determines constant simulation time and allocated computational resources), the radius of the spherical domain was kept equal to the wavelength, so both the elements and the overall domain volume were scaling in a similar way.

For the time-domain simulations, we made use of the "Electromagnetic Waves, Transient" physics interface, which solves for the time dependent vector potential. In this case, to model the air/crystalline quartz interface, we considered a square prism domain divided in two parts by the $z = 0$ plane. The prism height was 1100 μm (750 μm for the air domain, 350 μm for the crystalline quartz substrate), and the square base had a side length of 350 μm. The incident quasi-single-cycle terahertz pulse was injected from the upper boundary as a function of time, and the time-dependent solver took care of its propagation in the simulation domain, with a fixed time step of 5 fs. The electric field of the incident pulse was analytically modeled considering the second derivative of a Gaussian-shaped pulse [7], i.e.

$$E_{THz,y}(t) = E^0_{THz}\left(1 - \frac{t^2}{s^2_{THz}}\right)\exp\left(-\frac{t^2}{2s^2_{THz}}\right), \tag{S2}$$

where E^0_{THz} is the peak electric field, and $s_{THz} = 0.185$ ps is the standard deviation.

S3 Supplementary plots for the dragonfly antenna

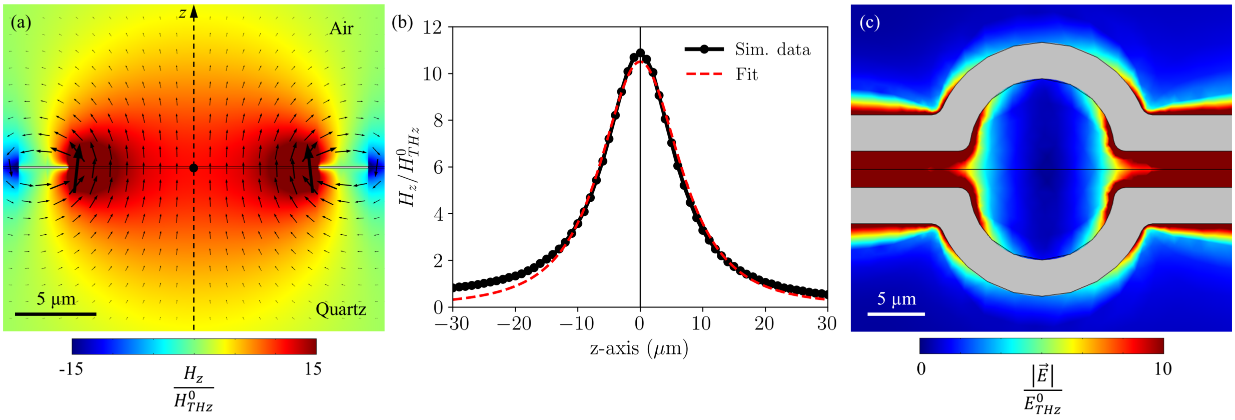

Figure S2: (a) Map of the out-of-plane magnetic field enhancement for the dragonfly antenna at 1.2 THz in a plane parallel to the yz-plane and passing through the center of the active region (marked by a black dot). The enhancement is calculated with respect to the H^0_{THz} amplitude of the incident field. The arrows show the (H_y, H_z) vectors. (b) Line profile of the out-of-plane magnetic field enhancement extracted along the black dashed line in panel (a). As a reference, the profile is fitted with the equation for the magnetic field generated along the axis of a circular current loop [8]: $H_z/H^0_{THz} = IR^2/\left[2H^0_{THz}\left(z^2+R^2\right)^{3/2}\right]$, where R is the loop radius, and I is the current. The fit procedure (least squares) returns $R = (9.8 \pm 0.2)$ μm, and $I/H^0_{THz} = (205 \pm 2)$ μm. (c) Map of the electric field enhancement for the dragonfly antenna at 1.2 THz in the $z = 0$ plane. The enhancement is calculated with respect to the E^0_{THz} amplitude of the incident field.

S4 Supplementary plots for the octopole antenna

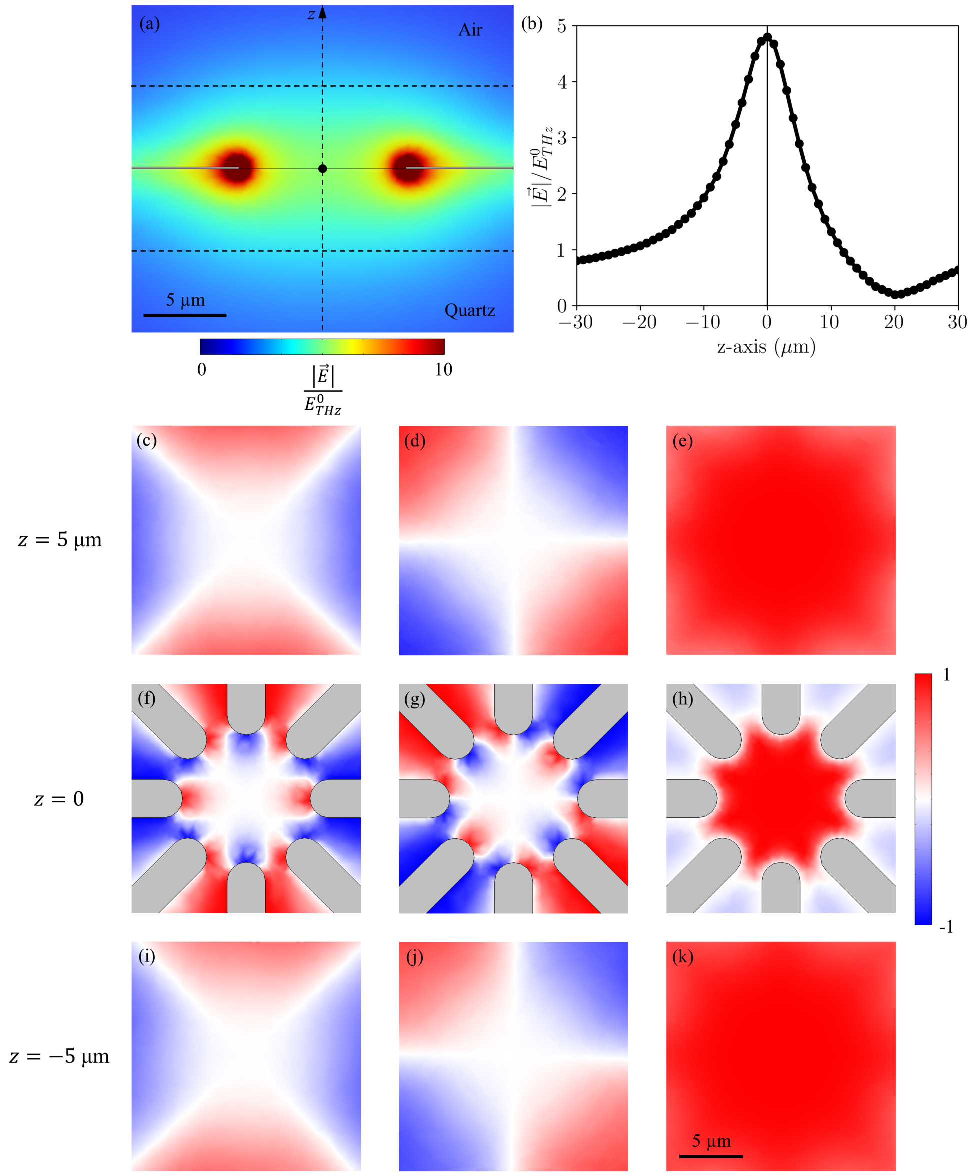

Figure S3: (a) Map of the electric field enhancement for the octopole antenna at 1.2 THz in a plane parallel to the xz-plane and passing through the center of the active region (marked by a black dot). The enhancement is calculated with respect to the E^0_{THz} amplitude of the incident field. (b) Line profile of the electric field enhancement extracted along the vertical black dashed line in panel (a). (c)-(k) Maps of the normalized Stokes parameters [9] at 1.2 THz, to quantify the polarization state in the active region. Panels (c), (d), and (e) show the map of S_1/S_0, S_2/S_0, and S_3/S_0 (respectively) in a plane parallel to the $z = 0$ plane and located 5 μm above the air/quartz interface, as indicated by the upper horizontal black dashed line in panel (a). Panels (f), (g), and (h) show the map of S_1/S_0, S_2/S_0, and S_3/S_0 (respectively) in the $z = 0$ plane. Panels (i), (j), and (k) show the map of S_1/S_0, S_2/S_0, and S_3/S_0 (respectively) in a plane parallel to the $z = 0$ plane and located 5 μm below the air/quartz interface, as indicated by the lower horizontal black dashed line in panel (a). While the S_0 parameter represents the total intensity, S_1 and S_2 are associated with linear polarization along two sets of orthogonal axes, and S_3 is associated with circular polarization.

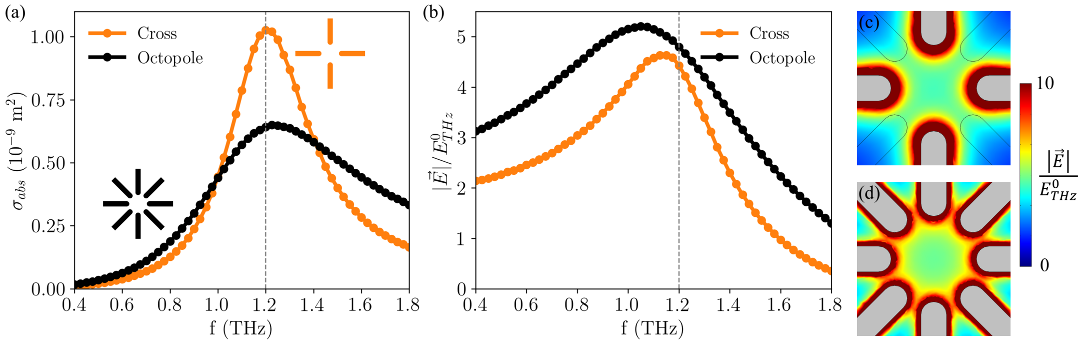

Figure S4: (a) Absorption cross sections calculated in the frequency domain for circularly-polarized radiation at normal incidence. Besides the octopole antenna (as already reported in Fig. 4(c) of the main text), the plot also shows the absorption cross section for the cross antenna obtained by removing the electrodes aligned at 45 degrees with respect to the x- and y-axis. (b) Total electric field enhancement at the center of the active region, calculated with respect to the incident field E^0_{THz}. Despite having similar enhancement factors at the target frequency of 1.2 THz, the octopole design is associated with a larger bandwidth than the cross antenna, as also visible in panel (a). (c),(d) Maps of the electric field enhancement (1.2 THz, $z = 0$ plane) for the cross and octopole antenna, respectively. In panel (c), the placement of the removed electrodes is shown.

References

[1] COMSOL Multiphysics® v. 6.1. `https://www.comsol.com`. COMSOL AB, Stockholm, Sweden.

[2] M. Savoini, S. Grübel, S. Bagiante, H. Sigg, T. Feurer, P. Beaud, and S. L. Johnson, "Thz near-field enhancement by means of isolated dipolar antennas: the effect of finite sample size," *Optics Express*, vol. 24, pp. 4552–4562, Mar 2016.

[3] J. Jin, *Theory and Computation of Electromagnetic Fields.* Wiley, 1st ed., Nov. 2010.

[4] C. L. Davies, J. B. Patel, C. Q. Xia, L. M. Herz, and M. B. Johnston, "Temperature-dependent refractive index of quartz at terahertz frequencies," *Journal of Infrared, Millimeter, and Terahertz Waves*, vol. 39, no. 12, pp. 1236–1248, 2018.

[5] Q.-Y. Wen, H.-W. Zhang, Y.-S. Xie, Q.-H. Yang, and Y.-L. Liu, "Dual band terahertz metamaterial absorber: Design, fabrication, and characterization," *Applied Physics Letters*, vol. 95, no. 24, p. 241111, 2009.

[6] D. M. Pozar, *Microwave Engineering.* Hoboken, NJ: John Wiley & Sons, Inc, 4th ed., 2012.

[7] M. C. Hoffmann and J. A. Fülöp, "Intense ultrashort terahertz pulses: generation and applications," *Journal of Physics D: Applied Physics*, vol. 44, no. 8, p. 083001, 2011.

[8] J. D. Jackson, *Classical Electrodynamics.* New York: Wiley, 3rd ed., 1999.

[9] H. C. van de Hulst, *Light Scattering by Small Particles.* New York: Dover Publications, Inc., 1981.
